# 基于代谢组学和蛋白组学研究砷暴露对肥胖孕鼠肝脏代谢分子网络的影响

**DOI:** 10.3724/SP.J.1123.2024.05028

**Published:** 2025-01-08

**Authors:** Lijing CAI, Yan WANG, Junfeng TAN, Haixia ZHOU, Shijia LIANG, Yan WU, Jie ZHANG

**Affiliations:** 1.厦门大学公共卫生学院, 福建 厦门 361102; 1. School of Public Health, Xiamen University, Xiamen 361102, China; 2.福建医科大学公共卫生学院, 福建 福州 350122; 2. School of Public Health, Fujian Medical University, Fuzhou 350122, China

**Keywords:** 砷, 肥胖, 孕鼠, 肝脏, 代谢组学, 蛋白组学, arsenic, obesity, pregnant mice, liver, metabolomics, proteomics

## Abstract

砷是普遍存在的环境毒物,可能影响生物体的正常生理过程。尽管已有研究探讨了砷对健康的影响,但砷暴露对肥胖孕妇肝脏代谢的影响及其作用机制尚不明确。本研究旨在通过高脂饮食诱导肥胖孕鼠模型,并采用灌胃方式模拟孕期砷暴露,以探究其对肝脏代谢的影响。孕鼠砷暴露后,我们利用代谢组学和蛋白组学技术,结合病理生化分析,对肝脏组织进行了深入研究。结果显示,砷暴露显著增加了肥胖孕鼠肝脏中的脂质累积,并伴随着炎症因子和氧化应激标志物的升高。代谢组学分析揭示了砷暴露对脂质代谢通路,特别是花生四烯酸代谢的显著影响。此外,蛋白组学分析确认了与脂质合成、氧化应激和炎症反应相关的蛋白质表达水平发生了变化。这些结果表明,砷暴露可能通过多种代谢途径和蛋白质调节通路,对肥胖孕鼠肝脏的脂质代谢产生显著影响。本研究不仅为理解砷暴露与肥胖及相关代谢性疾病之间的关系提供了新的科学视角,也为环境健康风险评估和公共卫生政策的制定提供了重要参考。

## Abstract (en)

Arsenic is a ubiquitous environmental toxin that can affect normal physiological processes. Although the health impacts of arsenic have been investigated, its influence on hepatic metabolism in obese pregnant women and the underlying mechanisms remain unclear. Multi-omics analysis, including metabolomics and proteomics, can improve the understanding of arsenic-induced hepatotoxicity in obese pregnant women. This study aimed to investigate the adverse effects of gestational arsenic exposure on hepatic metabolism in high-fat-diet-induced obese pregnant mice. Following arsenic exposure during pregnancy, the liver tissue was evaluated comprehensively using metabolomics and proteomics techniques combined with pathological and biochemical analyses.

Arsenic exposure not only significantly increased lipid accumulation in the livers of obese pregnant mice but also elevated inflammatory factors and oxidative stress markers. Specifically, histopathological examination revealed more steatosis, inflammatory cell infiltration, and hepatocyte ballooning in the livers of arsenic-exposed mice than in those of controls. These changes indicate that arsenic exposure exacerbates hepatic lipid accumulation and induces liver damage in the context of obesity. Metabolomic analysis provided further insight into the metabolic-level disruption caused by arsenic exposure. Significant changes were observed in lipid metabolism pathways, particularly the arachidonic acid metabolism pathway. As arachidonic acid and its metabolites play important roles in inflammation and oxidative stress, this pathway may be critical in arsenic-induced hepatotoxicity. Additionally, proteomic analysis showed differences in the expression levels of several key proteins involved in lipid synthesis, oxidative stress, and inflammatory response. Notably, oxidative-stress-related proteins, including glutathione peroxidase 4 (GPX4), were upregulated, suggesting an increased oxidative burden. In summary, there are complex interaction mechanisms among arsenic exposure, inflammatory response, and related lipid metabolism. The integration of metabolomics and proteomics aided in clarifying the molecular alterations induced by arsenic. The results show that arsenic exposure significantly affects hepatic lipid metabolism in obese pregnant mice through multiple metabolic pathways and protein regulatory mechanisms.

In addition to providing new insights into the relationship between arsenic exposure and obesity as well as related metabolic diseases, this study can act as a reference for environmental health risk assessment and the formulation of public health policies. This enhanced understanding of the adverse effects of arsenic on hepatic metabolism will contribute to the development of strategies for mitigating the health risks associated with environmental toxins, particularly for vulnerable groups such as obese pregnant women.

肥胖作为全球性的慢性代谢性疾病,与多种健康问题紧密相连,自1975年以来,患病率在全球范围内几乎增长了2倍,影响了约15%的成年女性^[1]^。在怀孕期间,女性的代谢系统会经历显著变化以适应胎儿的生长和发育需求,包括基础代谢率的提升、碳水化合物代谢的调整以及脂肪储存和蛋白质需求量的增加。孕期肥胖不仅会影响全身及局部的内分泌和炎症通路,还可能导致代谢过程紊乱,增加生殖和妊娠并发症的风险^[2]^。这种影响可能从女性围孕期开始直至胎儿成年期^[3]^。肝脏是关键的脂代谢和胰岛素作用的器官,相较于普通孕妇,肥胖孕妇的肝脏将承受更大的压力,表现为增强的胰岛素抵抗、肝酶水平的升高以及氧化应激和炎症水平的增加^[4]^。

环境污染物暴露会加剧孕妇体内的代谢紊乱。无机砷(iAs)是自然环境中常见的有害元素,人们通常通过饮用水和受污染的食物摄入^[5]^。研究表明,即使是低水平的砷暴露也会影响孕妇的脂代谢稳态,从而增加孕期无机砷暴露的风险^[6,7]^。肝脏是砷代谢的主要靶器官,砷对肝脏的毒性效应主要表现为氧化胁迫和炎症反应^[8,9]^,这与肥胖等代谢性疾病的发生密切相关^[10]^。对于体质量指数(BMI)较高的女性,无机砷暴露的影响尤为显著^[11]^,而且相比非孕期女性,砷对孕期肥胖女性的危害显著增加^[12]^。低浓度的砷可以通过增强炎症反应,加剧高脂肪饮食引起的肝损伤^[13]^,表明肥胖与砷之间可能存在相互作用。此外,慢性砷暴露叠加高脂饮食(HFD)显著增加小鼠的肝纤维化风险^[14]^。流行病学数据也显示,砷暴露会增加与肥胖相关的疾病——非酒精性脂肪肝(NAFLD)的患病风险^[15]^。上述研究提示,砷可能是加重肥胖引起肝损伤的潜在风险因素。鉴于肥胖和砷暴露都可能通过氧化胁迫和炎症反应等途径引起肝脏功能失调或损伤,确定它们的协同作用程度至关重要,因为这对饮用水和其他食物来源中砷的允许浓度产生至关影响。但是,截至目前,砷暴露与肥胖孕妇的肝脏功能受损之间的关系尚不明确,探究这一问题具有重大的现实意义。

代谢组学和蛋白组学能够全面、系统地分析生物体内的代谢物和蛋白质在环境胁迫下发生的全局性变化,是研究环境污染物毒性机理的有效工具^[16,17]^。本研究采用高脂饮食诱导肥胖孕鼠模型,合笼成功后,通过灌胃方式模拟孕期砷暴露的日常情况。暴露结束后,利用代谢组学和蛋白组学技术,结合病理生化结果对肝脏组织进行了深入分析。本研究将为深入理解砷暴露与肥胖及相关代谢性疾病之间的关系提供重要的科学依据,为预防和治疗这些疾病提供新的靶点和策略,也为公共卫生政策部门制定更有效的环境保护和健康促进措施提供依据。

## 1 实验部分

### 1.1 仪器、试剂与材料

Dionex液相色谱-Q-Exactive Orbitrap质谱联用系统和EASY-nLC 1000 system液相色谱-Q-Exactive Orbitrap Fusion质谱联用系统(美国Thermo Fisher Scientific公司); Zeiss AxiOscan 7显微镜玻片扫描系统(德国卡尔·蔡司股份公司); BS-240vet全自动生化分析仪(中国迈瑞生物医疗电子股份有限公司)。

亚砷酸钠(分析纯)和氨水购自上海阿拉丁生化科技股份有限公司;甲醇和乙腈为HPLC级,购自德国Merck公司;甲酸、丙酮和醋酸铵均为LC-MS级,购自美国Thermo Fisher公司;乙酸(LC-MS级)、十二烷基硫酸钠(SDS)和4-羟乙基哌嗪乙磺酸(HEPES)均购自美国Sigma-Aldrich公司;甘油三酯(TG)、高密度脂蛋白胆固醇(HDL-C)、低密度脂蛋白胆固醇(LDL-C)和总胆固醇(TC)测定试剂盒购自中国迈瑞生物医疗电子股份有限公司。

### 1.2 小鼠暴露实验

对7~8周龄的C57BL/6J雌性小鼠进行高脂肪饲料(HFD,含60%脂肪,Research Diets D12451)饲养4周以诱导肥胖。随后,这些肥胖雌鼠以2∶1的比例与喂食标准饲料的C57BL/6J雄性小鼠合笼。次日早晨,通过检查阴栓或阴道涂片中大量精子和动情期迹象来确定妊娠第0天(gestation day 0, GD0)。孕期通过灌胃方式模拟日常饮用水进行砷暴露,设置两组:肥胖对照组(超纯水)和肥胖暴露组。考虑到目前全球范围内70多个国家和地区接触的地下水砷含量远超指导值(≥0.1 mg/L),主要包括印度、孟加拉、柬埔寨、中国、越南、缅甸、美国等国家,其影响的人口达1.4亿,且饮用水中无机砷含量超过50~100 μg/L将对人体产生负面影响^[18⇓-20]^,本研究将砷暴露剂量设定为0.1 mg/L,并据此换算成相应的灌胃剂量:假设每人每日水摄入量为2 L,女性体重中位数为50 kg,则暴露剂量为0.004 mg/kg,已知人与动物暴露转化系数为10,即灌胃剂量为0.04 mg/kg。对照组灌胃相同体积的纯水。在孕期内,继续给予孕鼠高脂饲料,并记录其体重、外观和行为习性等参数。

妊娠第20天时,对孕鼠进行称重,然后处死,眼球采血,离心分离血清,随后利用全自动生化分析仪检测血清甘油三酯、总胆固醇、低密度脂蛋白胆固醇和高密度脂蛋白胆固醇水平。解剖肝脏组织,用生理盐水漂洗,滤纸吸干,称质量,计算肝脏系数。肝脏系数=肝脏质量/体重。

### 1.3 肝脏组织病理学和免疫组化(IHC)分析

切取部分肝脏样本浸泡在4%的多聚甲醛溶液中进行固定和苏木精-伊红(H&E)染色处理,以观察和评估细胞结构的变化。此外,对肝脏样本进行免疫组化染色,检测超氧化物歧化酶1(SOD1)和白细胞介素-6(IL-6)的表达水平,以评估体内的氧化应激反应和炎症状态。

### 1.4 肝脏蛋白质组分析

准确称取10 mg肝脏组织,利用4%的SDS和10 mmol/L的HEPES缓冲液(pH 8.0)匀浆提取肝脏蛋白质。使用二硫苏糖醇对蛋白质进行还原处理,随后用碘乙酰胺进行烷基化处理,丙酮沉淀。吸取少量蛋白质,经胰蛋白酶酶解成肽段,C_18_ StageTips微量移液管脱盐。

利用EASY-nLC液相色谱-Q-Exactive Orbitrap Fusion质谱联用系统进行分析。液相色谱柱为ReproSil-Pur C_18_纳流分析柱(200 mm×0.1 mm, 1.9 μm),流速为250 nL/min。流动相A: 0.1%甲酸水溶液,流动相B: 1%甲酸乙腈溶液。梯度洗脱程序:0~2 min, 3%B~7%B; 2~77 min, 7%B~22%B; 77~92 min, 22%B~35%B; 92~94 min, 35%B~90%B; 94~110 min, 90%B。质谱仪在数据独立采集(DIA)模式下采集蛋白组数据,DIA分析的扫描范围为*m/z* 395~1205,喷雾电压设置为2000 V, DIA工作流程包括以60000分辨率进行MS扫描,然后以30000分辨率进行连续的高能碰撞解离MS/MS扫描。MS/MS自动增益控制设置为10^5^,最大注入时间为55 ms,高能碰撞解离碎片的归一化碰撞能量设定为30%。使用DIA-NN软件(Data-Independent Acquisition with Neural Network)对蛋白组原始数据进行分析,蛋白质和肽段水平的错误发现率(FDR)阈值设置为0.01,其他参数保持默认设置。

### 1.5 非靶向肝脏代谢组分析

根据METDNA2的规范流程^[21]^进行非靶向肝脏代谢组分析。精密称取20 mg肝脏组织,加入200 μL纯水,低温均质,再加入800 μL甲醇-乙腈(50∶50, v/v),涡旋,水浴超声,4 ℃孵育1 h,以13000 r/min离心15 min,吸取上清液,离心浓缩至近干,再用100 μL乙腈-水(50∶50, v/v)重溶冻干样品,离心取上清液转移至进样瓶。

利用Dionex液相色谱-Q-Exactive Orbitrap MS联用系统进行非靶向肝脏代谢组分析。液相色谱柱为Waters UPLC BEH Amide色谱柱(100 mm×2.1 mm, 1.7 μm),流速为0.3 mL/min。流动相A:含25 mmol/L醋酸铵和25 mmol/L氢氧化铵的水溶液,流动相B:乙腈;梯度洗脱程序:0~1 min, 95%B; 1~14 min, 95%B~65%B; 14~16 min, 65%B~40%B; 16~18 min, 40%B; 18~18.1 min, 40%B~95%B; 18.1~23 min, 95%B。进样量为2 μL,色谱柱温度保持25 ℃,自动进样器温度保持4 ℃。质谱仪配有电喷雾电离(ESI)源,在正、负离子同时扫描模式下采集一级质谱数据,扫描范围为*m/z* 70~1000。喷雾电压设置为3500 V,探针加热器温度为425 ℃,毛细管温度为300 ℃,在数据依赖采集模式(DDA)下采集二级质谱数据。利用Met4DX软件处理代谢组图谱生成多元数据矩阵,参数设置:峰值检测CentWave,峰宽5~40 s,质量范围70~1200 Da,信噪比阈值10,峰对齐obiwarp,峰分组density,质量容差10^-6^,保留时间容差15 s,基于原始质谱数据填补缺失值。

### 1.6 数据处理与统计分析

#### 1.6.1 蛋白组学分析

首先使用SIMCA-P 14.1软件(Umetrics,瑞典)进行无监督主成分分析(PCA),评估分析系统的稳定性,然后进行正交偏最小二乘判别分析(OPLS-DA),区分对照组和暴露组。随后,采用LIMMA软件包筛选差异蛋白,筛选标准:变量差异贡献度(VIP) >1,倍数变化(FC)>1.5或<0.67以及*p*值<0.05。采用Cluster profiler对差异蛋白进行KEGG和GO富集分析,利用String数据库(https://string-db. org)构建差异蛋白质相互作用(PPI)网络,使用Cytoscape软件(版本3.9.1)进行可视化。

#### 1.6.2 代谢组学分析

使用PCA分析评估分析模型的稳定性,然后进行OPLS-DA分析区分对照组和暴露组。差异代谢物的标准:VIP >1, FC >1.5或<0.67以及*p*值<0.05。使用MetaboAnalyst代谢分析平台进行KEGG通路富集。

## 2 结果与讨论

### 2.1 砷暴露对肥胖孕鼠体重变化系数、肝脏系数及血清生化指标的影响

如图1a所示,对照组和暴露组孕鼠的体重变化系数和肝脏系数无明显差异。采用生化分析仪检测砷暴露对肥胖孕鼠脂质代谢的影响,包括TG、LDL-C、TC、HDL-C。如图1b所示,砷暴露组孕鼠的血清TG、TC和LDL-C浓度呈下降趋势,HDL-C浓度存在上升趋势,但结果无统计学差异。

**图1:**
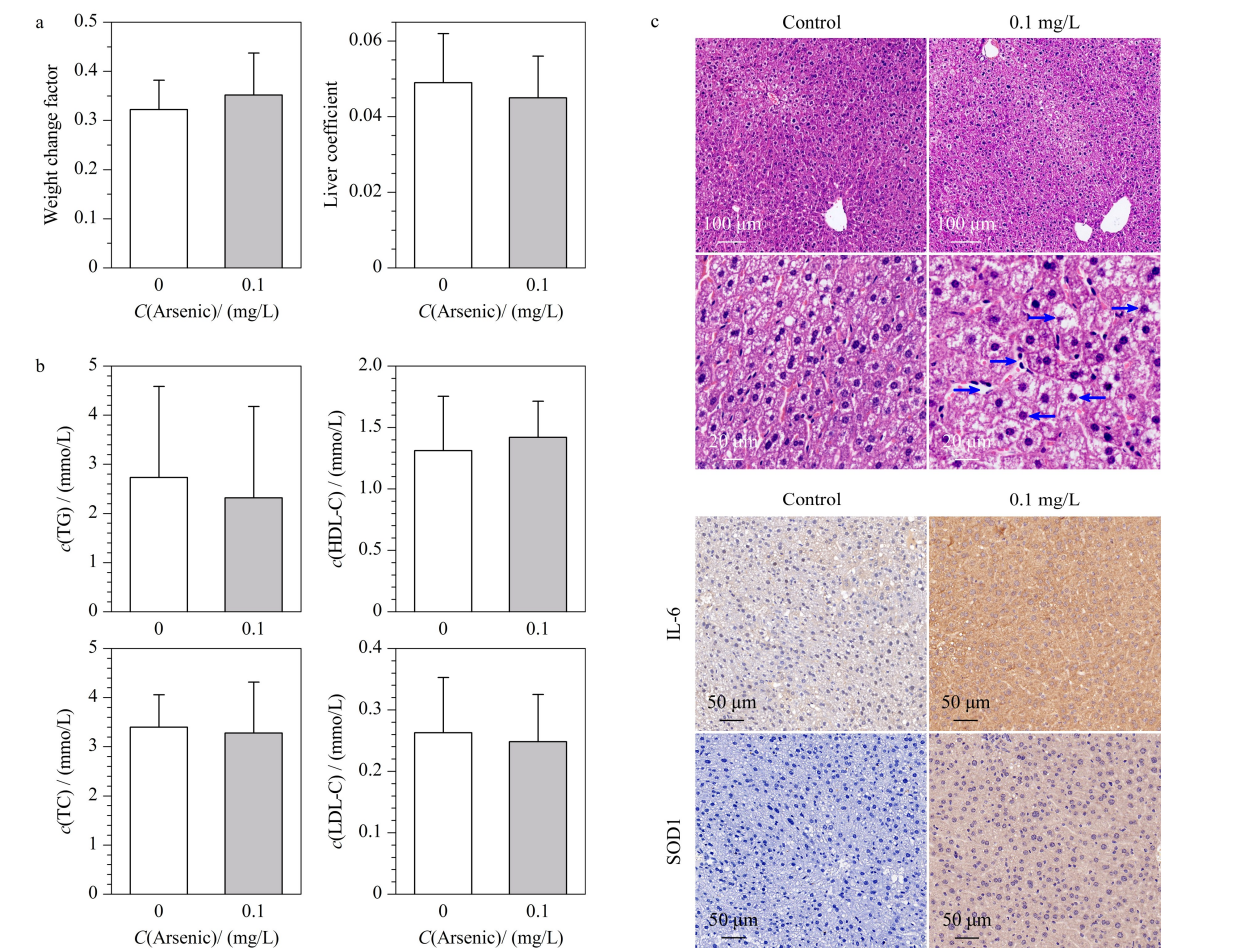
砷暴露对(a)肥胖孕鼠体重变化系数、肝脏系数(*n*=11)与(b)血清生化指标(*n*=11)的影响以及(c)砷暴露小鼠肝脏组织病理染色和免疫组化染色结果

### 2.2 砷暴露对肥胖孕鼠肝脏组织病理和免疫组化的影响

如图1c所示,对照组孕鼠肝小叶结构完整,无变性、坏死、炎性细胞浸润及纤维组织增生,同时未出现肝脂肪变性。相比之下,暴露组孕鼠出现明显的肝脂肪变性(如箭头所示方向),表明妊娠期砷暴露导致肝脏脂质积累及炎细胞浸润。对肝脏组织进行IHC染色(IL-6和SOD1),如图1c所示,暴露组孕鼠肝脏组织中IL-6免疫反应性显著增强,提示炎症反应的发生。同时,暴露组的SOD1阳性表达表明砷暴露引起孕鼠肝脏组织的氧化胁迫。这些结果表明砷暴露导致了肥胖孕鼠的肝脏损伤。

### 2.3 砷暴露对肥胖孕鼠肝脏蛋白质组的影响

如OPLS-DA模型所示(见图2a),对照组和暴露组的样本点投影在各自的区域,模型的稳健性通过999次置换检验得到验证,支持了我们的聚类分析具有很高的可靠性,表明砷暴露导致孕鼠肝脏蛋白组发生了显著的整体变化。我们进一步鉴定到161个差异蛋白质(见图2b),主要定位于细胞器膜、核糖体、肽酶复合物等结构中,在生物过程中主要参与马富酸、血红素、卟啉类化合物、二羧酸、核糖核苷酸等多种代谢途径(见图2c)。在分子功能方面,这些蛋白质与氧化还原酶、水解酶及裂解酶活性密切相关,并在羧酸、有机酸及维生素的结合中发挥作用。KEGG通路分析(见图2c)进一步指出,这些差异蛋白在过氧化物酶体增殖物激活受体(PPAR)信号通路、碳代谢、氨基酸生物合成等关键代谢通路中显著富集,表明砷暴露可能影响肥胖孕鼠肝脏组织的多条代谢通路。

**图2:**
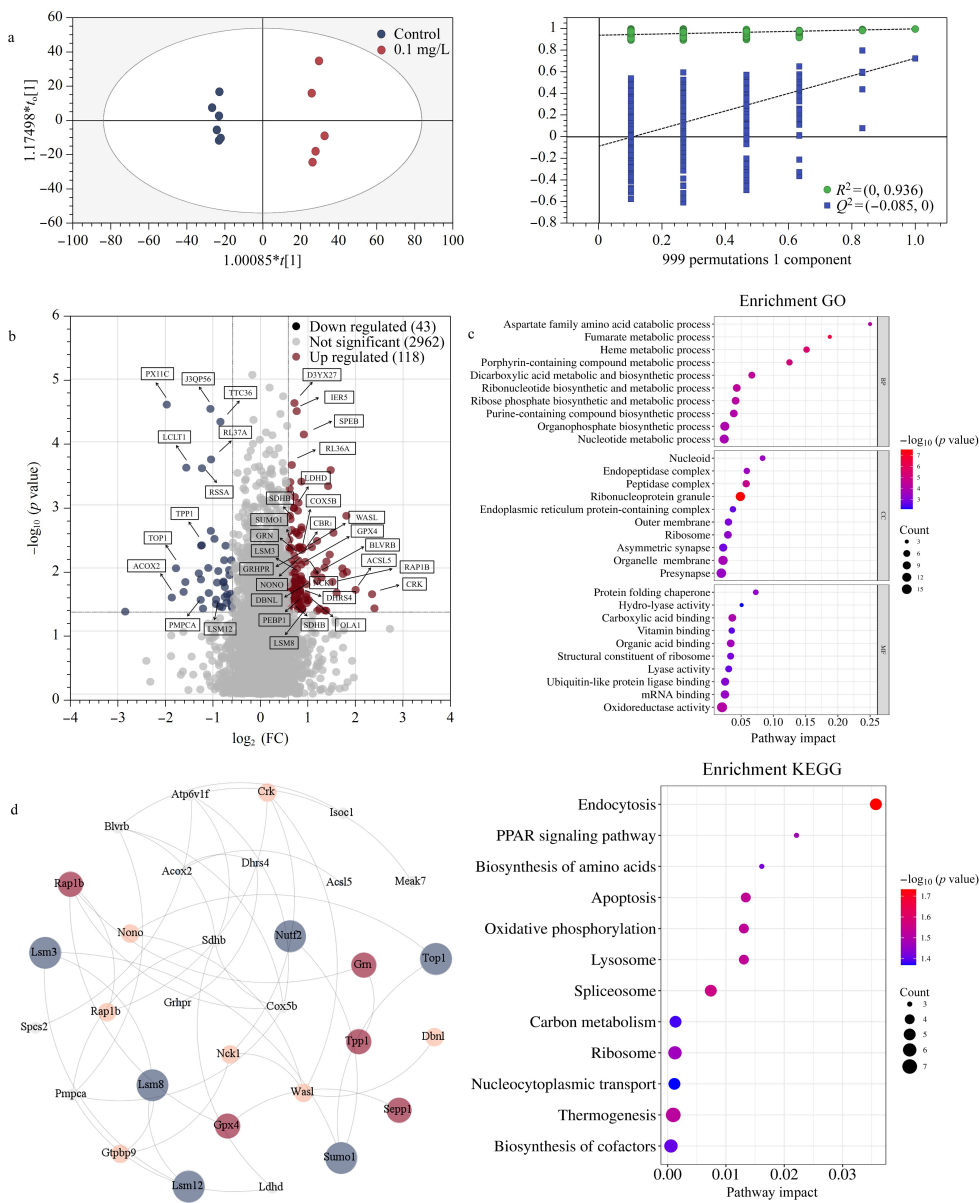
(a)肝脏蛋白组的OPLS-DA及999次置换检验结果,(b)对照和砷暴露组差异蛋白的火山图,(c)差异蛋白质的富集分析结果,(d)蛋白相互作用网络分析结果

为了探索这些差异蛋白之间的相互作用及潜在的生物学意义,我们利用STRING数据库构建了PPI网络,并对网络中的节点进行了详细的功能注释。PPI网络分析显示,RNA代谢相关蛋白(如Lsm3、Lsm8和Lsm12)显示出较强的相互作用(见图2d),提示他们在砷暴露影响下的肥胖孕鼠肝脏组织中可能具有重要的生物学功能。此外,网络中还识别出一些关键蛋白,例如谷胱甘肽过氧化物酶4(GPX4),这些蛋白在免疫调节、氧化应激和急性炎症反应等生物学过程中起着至关重要的作用。

### 2.4 砷暴露对肥胖孕鼠肝脏代谢组的影响

与蛋白组学研究结果类似,OPLS-DA模型也成功地区分了暴露组与对照组的肝脏代谢组(见图3a),模型的稳健性通过999次置换检验得到验证,支持了我们的聚类分析具有很高的可靠性。砷暴露胁迫下,孕鼠肝脏产生了95个显著变化的代谢物(见图3b),主要涉及脂质和脂类分子、有机酸及其衍生物、糖代谢相关化合物以及多种氨基酸及其衍生物。特别是脂质相关代谢物的变化最为显著,并且大多数呈现出表达下调的趋势(见图3b和图3c)。此外,通过KEGG富集分析,我们发现这些差异代谢物涉及49条代谢途径,包括花生四烯酸(AA)代谢、嘌呤代谢、一碳代谢等途径(见图3d)。其中,花生四烯酸代谢途径的改变尤其显著,指示了砷暴露可能影响一些关键的脂质代谢途径。

**图3:**
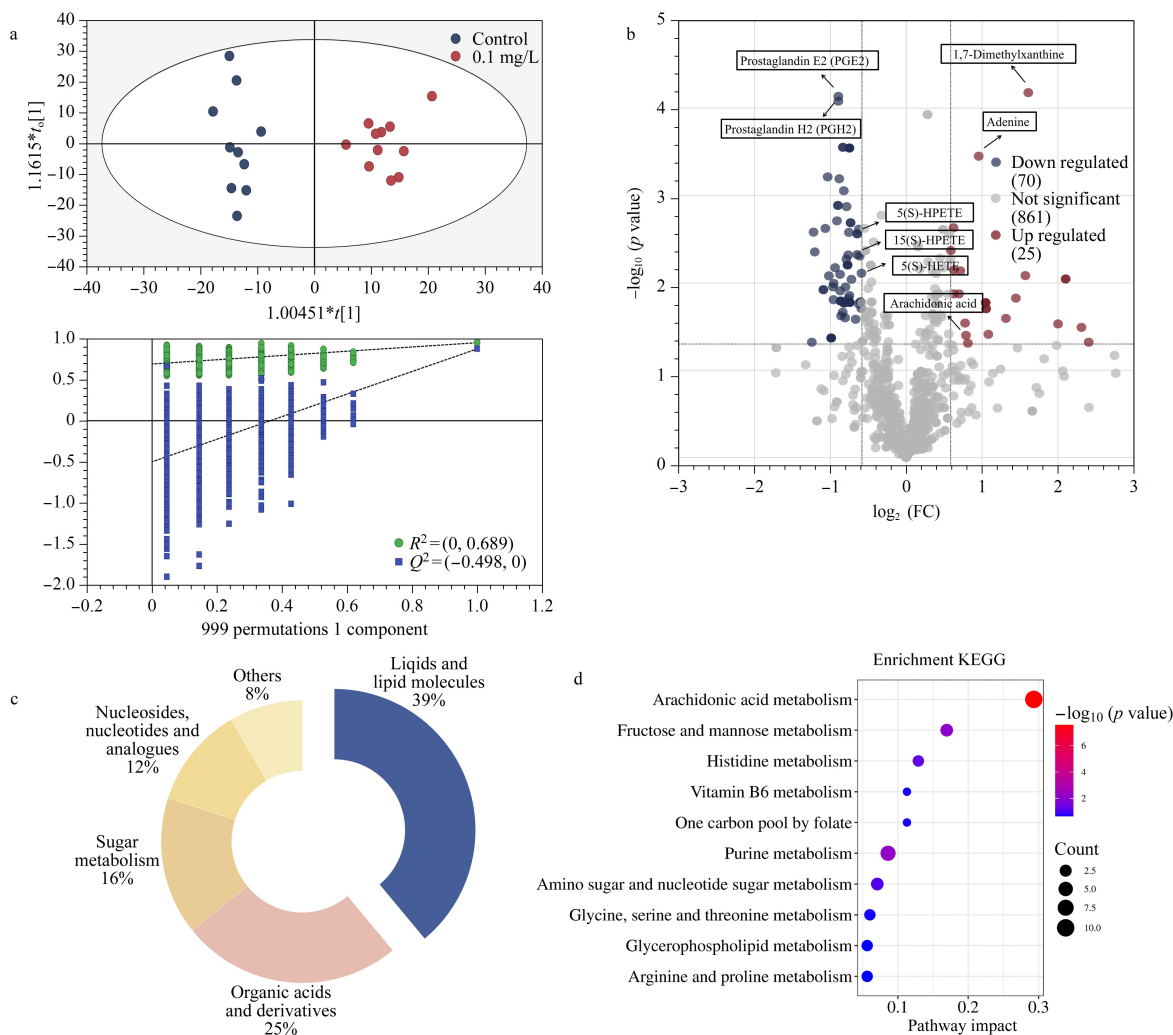
(a)肝脏代谢组的OPLS-DA及999次置换检验结果,对照组和砷暴露组差异代谢物的(b)火山图及(c)分类饼图,(d)差异代谢物的KEGG富集结果

### 2.5 砷暴露对肥胖孕鼠肝脏代谢分子网络的影响

砷对人体健康的影响已成为全球关注的重要公共卫生问题。流行病学研究揭示,人体摄入砷会引发肝脏代谢紊乱。本研究选取妊娠肥胖小鼠作为模型,通过0.1 mg/L砷暴露剂量,模拟妊娠期肥胖妇女这一敏感人群的砷暴露情况,旨在分析和探讨砷暴露对肥胖孕鼠肝脏代谢稳态的影响。结合组织切片、血清生化指标和多组学分析结果,我们发现砷暴露明显改变了肥胖孕鼠肝脏的分子代谢网络。

#### 2.5.1 砷暴露诱导肥胖孕鼠肝脏脂肪蓄积和炎症加重

本研究在雌性C57B/6J小鼠GD0时(即妊娠开始时)开始进行砷暴露,一直持续到GD20。结果显示,砷暴露组的雌鼠体重增长无显著差异性。然而,一些研究表明,较高剂量的砷暴露(如50 mg/L)会导致孕鼠体重增长减缓^[22⇓-24]^。这表明,不同的暴露剂量会引起不同的体重变化效应,而不同的小鼠品系与暴露方式等因素也会对孕鼠体重产生不同的影响。

肝脏是砷的主要代谢器官^[25]^,在本研究中,虽然肥胖孕鼠肝脏系数无显著变化,但组织病理学和免疫组化结果显示砷暴露会诱发肝脏组织的氧化胁迫和炎症反应,并加重肝损伤,具体表现为严重的炎症、脂肪变性、肝细胞变性和轻度纤维化。既往研究表明,无机砷长期暴露会导致青春期雄性小鼠及成年雄性大鼠产生肝脏病变,包括炎症和氧化损伤、脂肪堆积和肝纤维化^[26,27]^。在高脂饮食和砷联合处理的情况下,肝脏组织的脂质含量和氧化应激加重^[28]^,且砷通过炎症反应增强了高脂饮食诱导的肝损伤^[29]^,这与我们免疫组化的结果一致。流行病学研究也发现,尿砷水平与NAFLD呈正相关,长期暴露于含砷饮用水会增加肝硬化和肝细胞癌的风险^[30]^。另外,肝脏也是负责合成和释放内源性TG的主要器官,既往研究发现,高脂饮食喂养小鼠的肝脏会增加TG血液分泌以防止脂质在该器官中过度积聚,而砷暴露会抑制TG血液分泌从而导致TG在肝脏、肌肉和胰腺等非脂肪组织中的异常沉积^[31]^。本研究中,我们首次观察到暴露组孕鼠肝脏组织的脂肪堆积以及血清TG水平的非显著性下降。这一发现与以往针对雄性大鼠的砷暴露研究结果相似,后者显示砷暴露显著降低了血浆中TG和LDL-C的浓度^[32]^。由于男性和女性的TG代谢存在显著的性别差异,女性的TG水平普遍低于男性,且女性的血浆TG清除率更高^[33,34]^,这可能意味着砷暴露对雄性小鼠TG水平的影响程度较雌性更为显著。这些发现不仅强调了在肥胖和妊娠期砷暴露的背景下,脂质代谢紊乱是如何加剧肝脏损伤的,也为理解砷暴露对特定人群(如孕妇)健康影响的复杂性提供了新的视角。

#### 2.5.2 砷暴露引起肥胖孕鼠肝脏花生四烯酸代谢分子网络紊乱

代谢组学分析发现,砷暴露引起肥胖孕鼠肝脏花生四烯酸代谢通路的显著变化。AA及其衍生物在肝脏疾病的发生和发展中起着关键作用^[35]^。AA代谢有3种主要的酶促途径:环加氧酶(COX)、脂加氧酶(LOX)和细胞色素P450单加氧酶(P450)途径。这些途径产生多种脂质介质,如前列腺素(PG)、白三烯、羟基二十碳四烯酸(HETE)、环氧二十碳烯酸(EET)^[36]^。这些代谢物在炎症过程中发挥重要作用,如COX2衍生的促炎脂质介质前列腺素2(PGE2)与肝细胞脂质代谢相互作用,引起大鼠肝脏脂肪变性及炎症反应^[37]^。CYP450家族的酶参与抗炎EET和HETE的产生,使小鼠出现脂肪性肝炎^[38]^。此外,患有NAFLD的小鼠和人都表现出5-LOX通路的活性显著增加,并且5-LOX衍生物的表达增强与肝病的严重程度相关^[39,40]^。这些慢性炎症可以影响脂肪组织和肝脏的脂质代谢,进而影响TG水平。

本研究中,我们发现HETEs(如15-羟基过氧化二十碳四烯酸(15-HPETE)、5-HPETE)和PGs(如前列腺素 H2(PGH2)和PGE2)等AA代谢物质表达下调(见图4)。砷暴露激活了AA代谢的COX和LOX途径,引起15-HPETE表达显著下调,进而导致其下游产物5-HPETE表达下降。5-HPETE和15-HPETE负责白三烯和脂蛋白的产生,后者具有抗炎作用^[41]^。结合病理组织切片观察到的显著炎症反应,我们推测砷暴露在孕鼠肝脏组织中抑制了HPETE类代谢物的表达,并降低了脂蛋白的抗炎作用,从而导致了更加剧烈的炎症反应。PGs是由AA通过COX产生的生物活性脂质介质^[36]^。代谢组结果显示,暴露组中PGH2的表达显著下调,从而引起其下游产物PGE2表达进一步下调。既往研究表明,PGE2能够抑制超氧化物歧化酶1的表达或活性,导致细胞内超氧自由基水平升高,从而影响细胞的氧化还原平衡^[42]^。我们的免疫组化切片结果显示,暴露组表现出更强的氧化胁迫反应。砷暴露激活孕鼠肝脏AA代谢的COX通路,使PGE2表达下调,从而导致肝脏脂质代谢紊乱和氧化应激。另一项研究也表明,在妊娠期到哺乳期间对雌性大鼠进行砷暴露会增加体内活性氧类(ROS)水平,进而引发氧化应激^[43]^。尽管这些研究在暴露时间和研究样本上可能存在差异,但共同的发现强调了AA代谢在砷暴露反应中的关键作用。

我们在肝脏蛋白组分析中发现,抗氧化酶谷胱甘肽过氧化物酶4是差异蛋白PPI网络的关键节点。GPX4对于调节细胞内过氧化物质水平、炎症信号通路和免疫反应至关重要^[44]^,而且可通过抑制AA及其下游代谢氧化物(如5-HETE)的生成从而促进炎症消退^[36]^。

此外,考虑到GPX4作用的代谢产物HETE被认为是PPARα和PPARγ的潜在配体^[45]^,这与砷暴露显著影响PPAR信号通路的蛋白质组学KEGG结果相呼应。PPARs通过调节多种脂质代谢相关基因的转录来影响脂质代谢,包括脂肪酸氧化、脂质储存和血脂水平调节等,花生四烯酸作为一种重要的脂质分子,在这些过程中起着核心作用^[45]^。PPARs的激活可以通过调节炎症相关基因的表达,通过抑制炎症信号通路发挥抗炎作用。急性砷暴露的实验结果显示,小鼠血清中的5-HETE水平呈剂量依赖性增加^[44]^,这可能是因为急性砷暴露触发了AA代谢通路,从而引发炎症反应。这一现象与长期砷暴露导致的GPX4表达上调形成对比,后者通过促进5-HETE的消耗,展示出一种抗炎反应。这一发现强调了长时间砷暴露可能通过调节特定蛋白表达来激活抗炎机制,尤其是在肥胖孕鼠模型中。此外,GPX4和PPAR信号通路之间的相互作用突显了砷暴露影响脂质代谢和炎症反应的复杂机制,为进一步研究提供了重要线索。

**图4:**
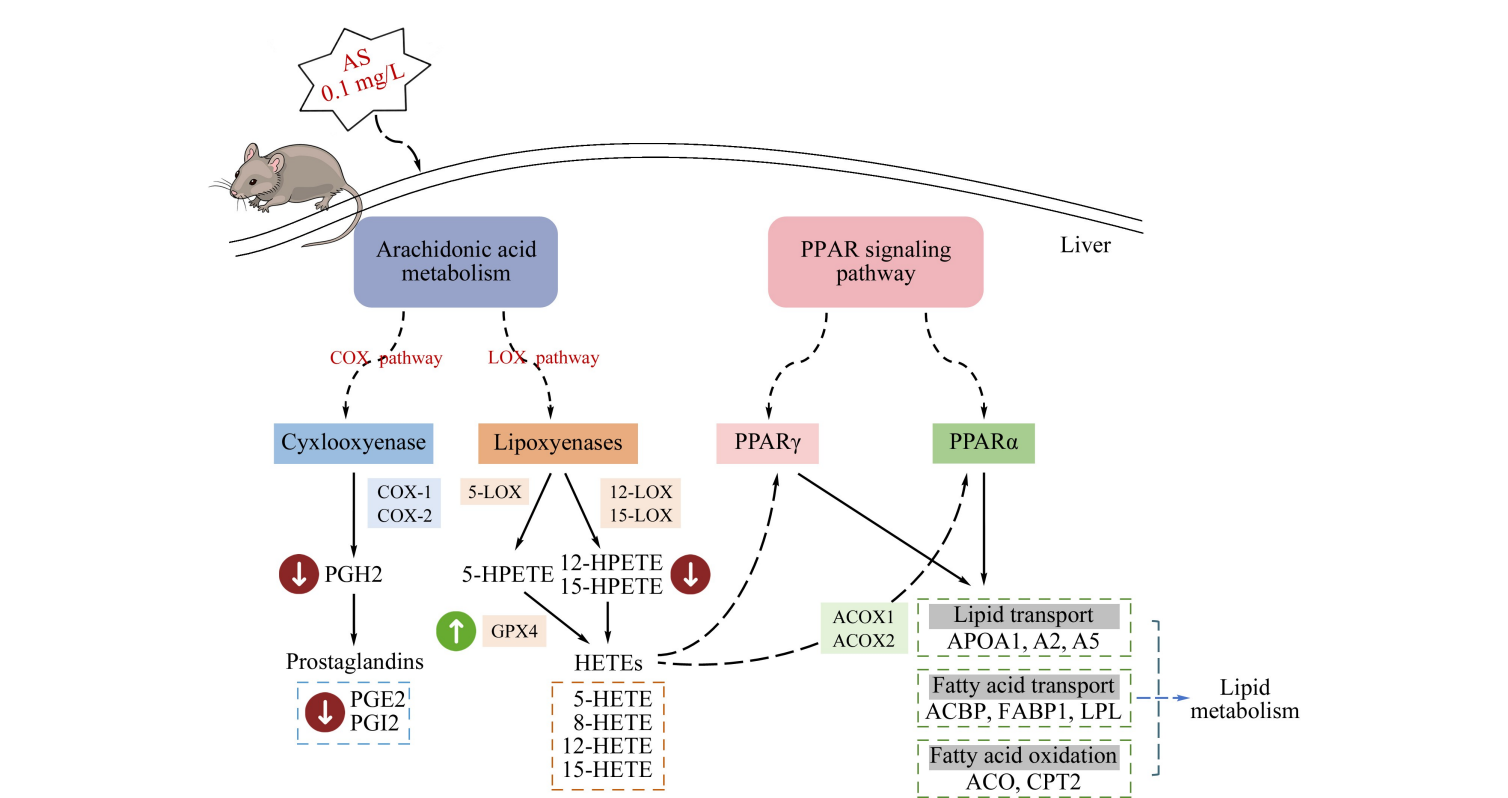
砷暴露影响肥胖孕鼠肝脏的脂质代谢通路

## 3 结论

本研究选取妊娠小鼠作为模型,通过0.1 mg/L砷暴露剂量,模拟妊娠期妇女的砷暴露情况,揭示了砷暴露通过多种代谢途径和蛋白质调节通路,对肥胖孕鼠肝脏的脂质代谢产生显著影响,为深入理解砷暴露与肥胖及相关代谢性疾病之间的关系提供了重要的科学依据,也为环境健康风险评估和公共卫生政策的制定提供了重要参考。
